# Supplementary figures and images for: RatXcan: A framework for cross-species integration of genome-wide association and gene expression data
Source: PLoS Genet. 2025 Mar 31;21(3):e1011583. doi: 10.1371/journal.pgen.1011583 (PMC12052193; doi:10.1371/journal.pgen.1011583)

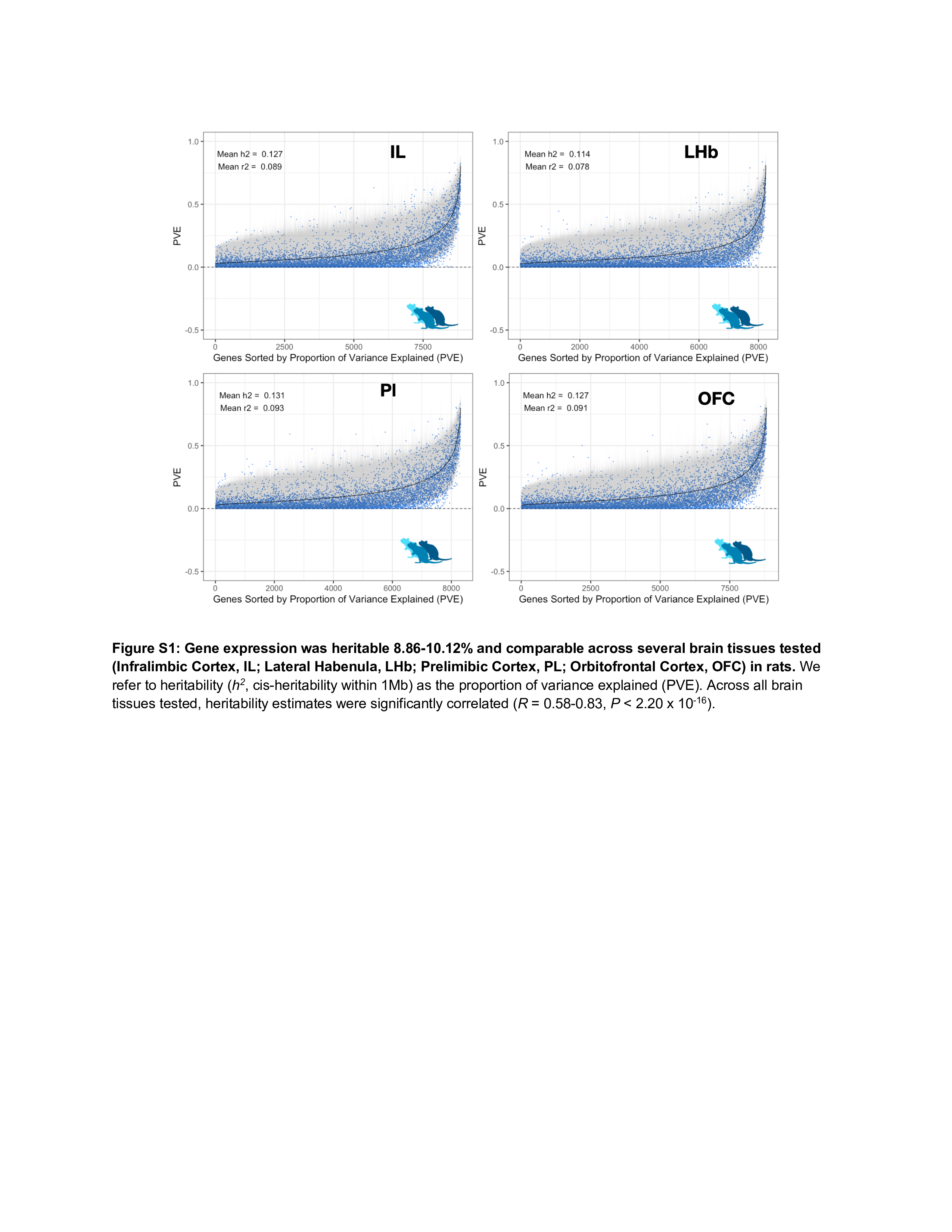

Supplement: S1 Fig — We refer to heritability (h2, cis-heritability within 1Mb) as the proportion of variance explained (PVE). Across all brain tissues tested, heritability estimates were significantly correlated (R = 0.58-0.83, P = 3.14 x 10^-19^). (TIFF) [file pgen.1011583.s001.tiff]

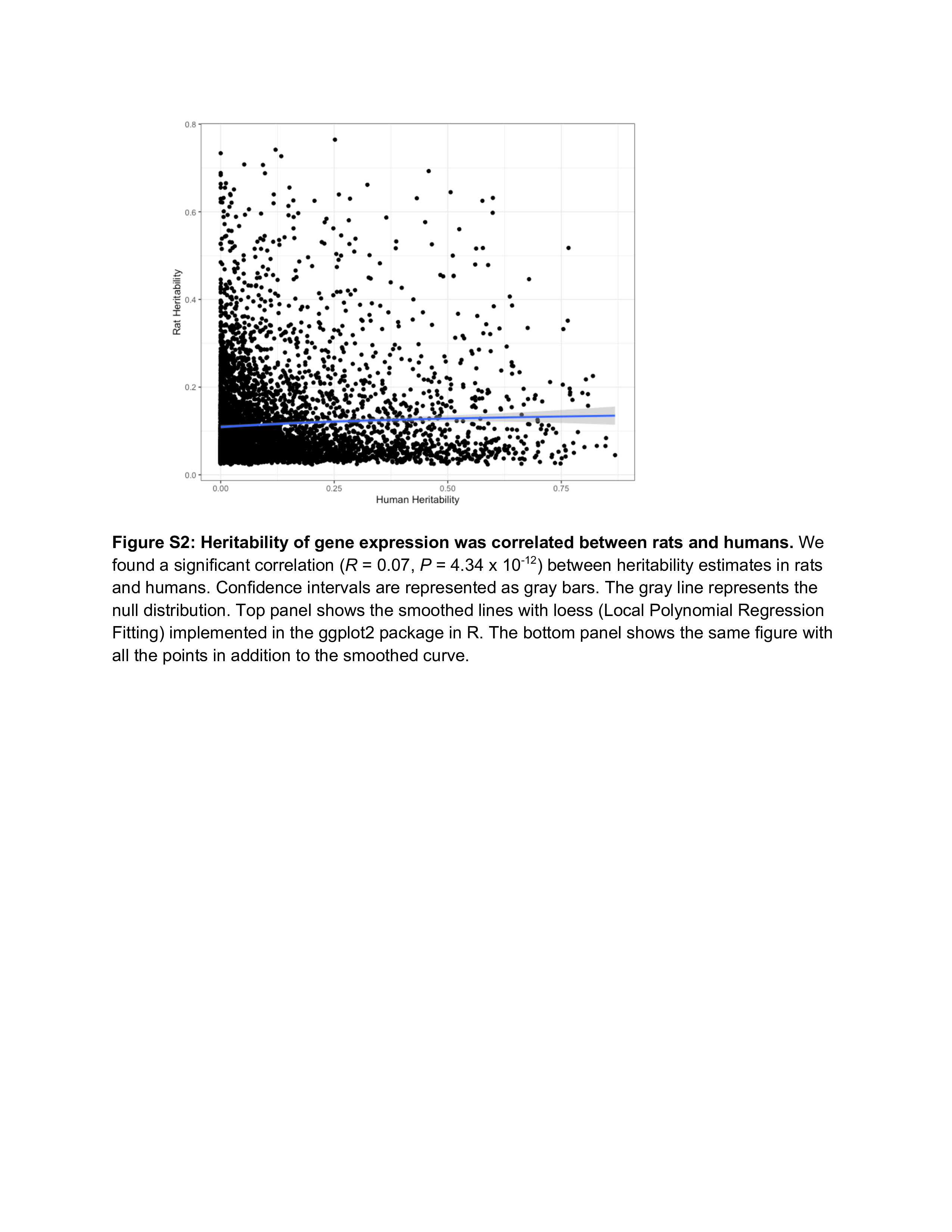

Supplement: S2 Fig — We found a significant correlation (R = 0.07, P = 4.34 x 10^-12^) between heritability estimates in rats and humans. Confidence intervals are represented as gray bars. The gray line represents the null distribution. Top panel shows the smoothed lines with loess (Local Polynomial Regression Fitting) implemented in the ggplot2 package in R. The bottom panel shows the same Fig with all the points in addition to the smoothed curve. (TIFF) [file pgen.1011583.s002.tiff]

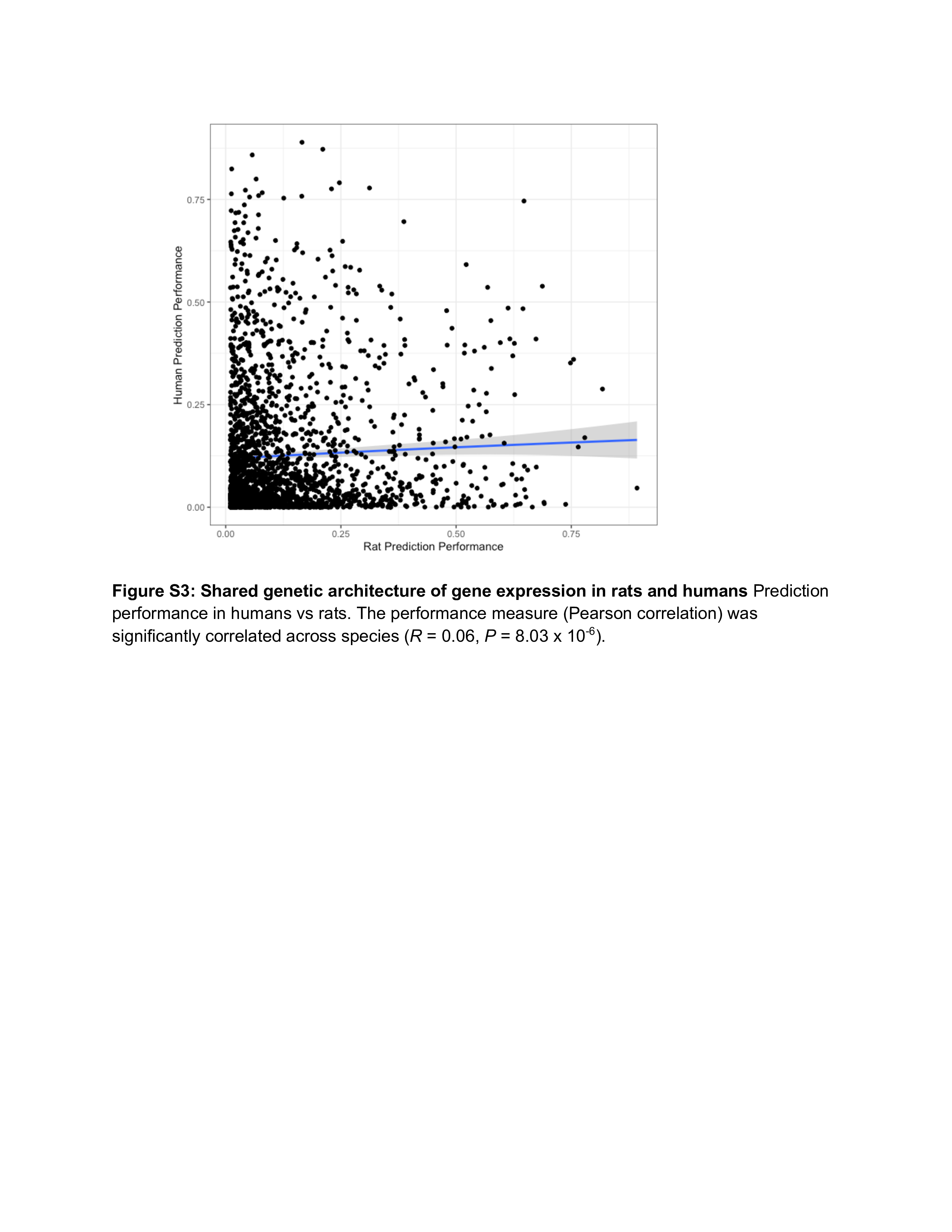

Supplement: S3 Fig — The performance measure (Pearson correlation) was significantly correlated across species (R = 0.06, P = 8.03 x 10^–6^). (TIFF) [file pgen.1011583.s003.tiff]

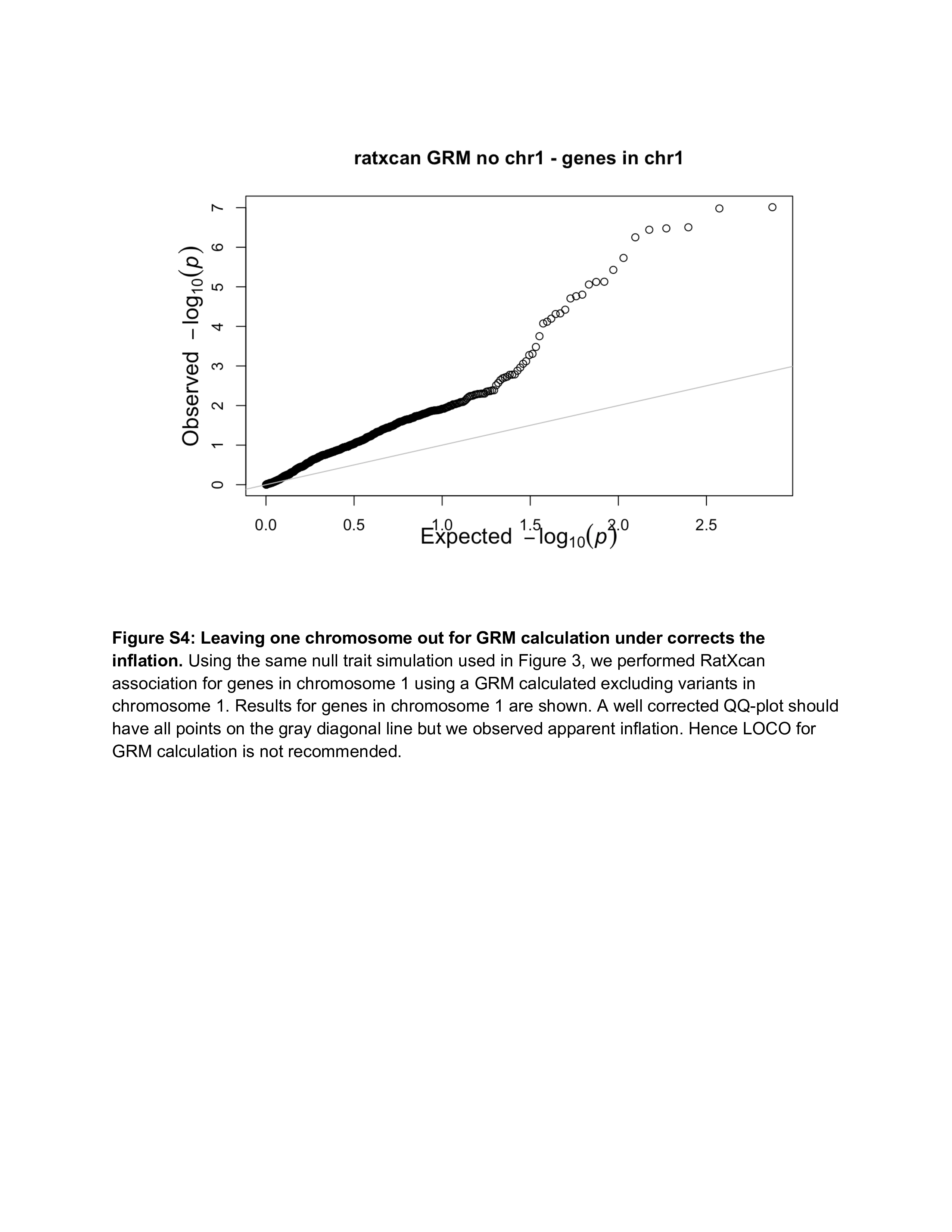

Supplement: S4 Fig — Using the same null trait simulation used in Fig 3, we performed RatXcan association for genes in chromosome 1 using a GRM calculated excluding variants in chromosome 1. Results for genes in chromosome 1 are shown. A well corrected QQ-plot should have all points on the gray diagonal line but we observed apparent inflation. Hence LOCO for GRM calculation is not recommended. (TIFF) [file pgen.1011583.s004.tiff]

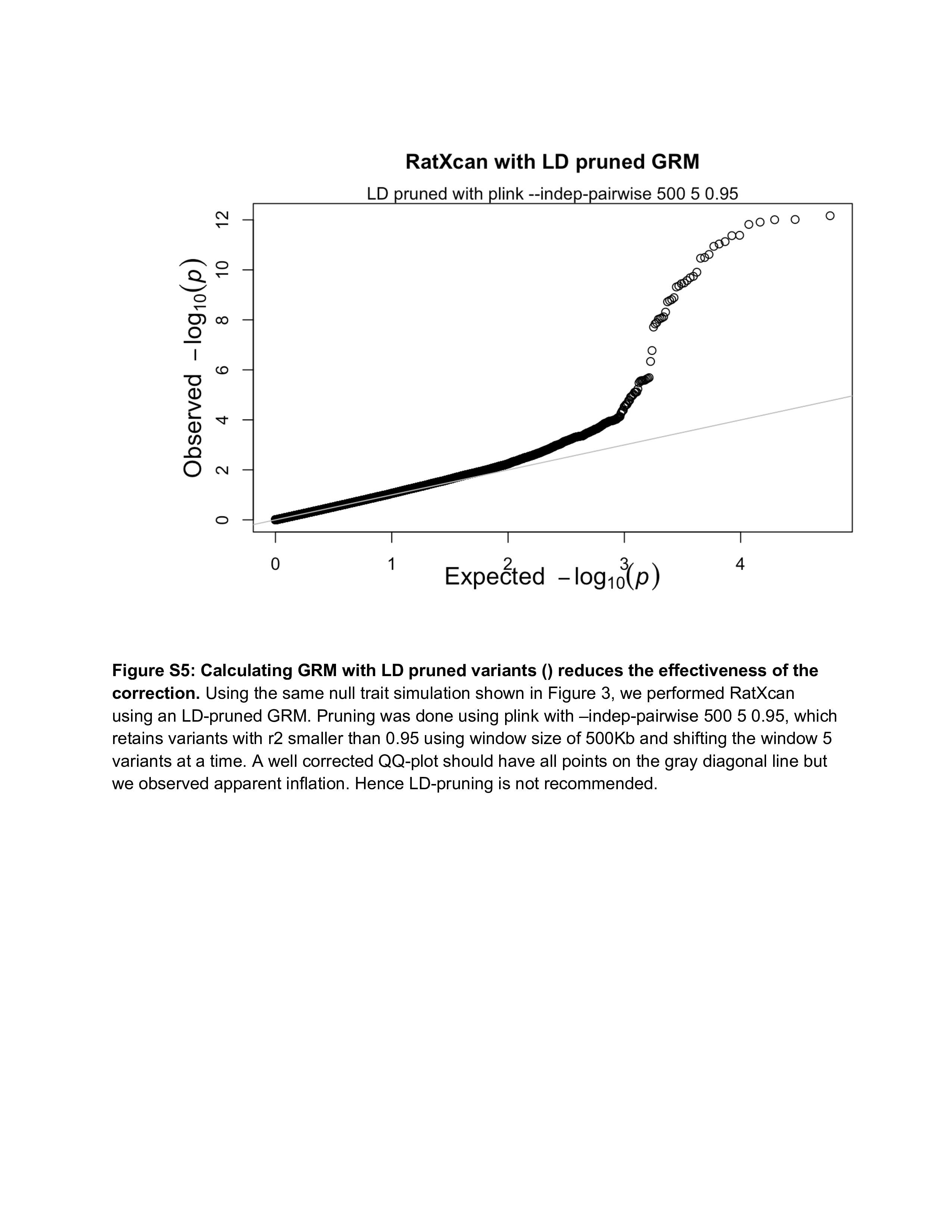

Supplement: S5 Fig — Using the same null trait simulation shown in Fig 3, we performed RatXcan using an LD-pruned GRM. Pruning was done using plink with –indep-pairwise 500 5 0.95, which retains variants with r2 smaller than 0.95 using window size of 500Kb and shifting the window 5 variants at a time. A well corrected QQ-plot should have all points on the gray diagonal line but we observed apparent inflation. Hence LD-pruning is not recommended. (TIFF) [file pgen.1011583.s005.tiff]
