# Supplementary material for: RatXcan: A framework for cross-species integration of genome-wide association and gene expression data
Source: PLoS Genet. 2025 Mar 31;21(3):e1011583. doi: 10.1371/journal.pgen.1011583 (PMC12052193; doi:10.1371/journal.pgen.1011583)
Supplement: Attachment — Submitted filename: Resposes-to-Review-for-RatXcan-PGENETICS-D-24-00652.pdf [file pgen.1011583.s009.pdf]

Reviewer's Responses to Questions

Reviewer #1

Santhanam et al. present RatXcan, a framework for PrediXcan-like analysis with samples of related individuals, demonstrating its utility for cross-species integration of genome-wide association and gene expression data. The manuscript is engaging and well-structured. While the study focuses on model organisms, as an animal quantitative geneticist, I believe RatXcan has significant potential for GTEx studies in farm animals. I have several minor comments and questions that may help broaden the study's impact:

1. Heritability and prediction models: The study uses variants within ±1Mb windows up- and downstream of each gene's transcription start and end sites for heritability estimation and prediction modeling, without accounting for relatedness using a GRM. The manuscript has well justified the use of GRM in RatXcan associations. I wonder if modeling relatedness is also necessary for prediction and cis-heritability estimation. In such cases, could a GRM be constructed excluding the variants within the ±1Mb window?

Response: The relatedness of the expression dataset was investigated in our prior publication (Munro et al 2022). In that study, we found that there was essentially no difference between using a linear model and a linear mixed model when considering cis-eQTLs because the animals in the expression data were chosen to be distantly related. We have pasted the relevant supplemental figure from that paper below. We have now added the following to the manuscript noting this and referring to Munro et al (2022).

"We did not use a linear mixed model (LMM) because in a prior publication (Munro et al 2022) we demonstrated that there was essentially no difference between using a linear model and an LMM for mapping cis-eQTLs in this dataset, perhaps in part because the rats were chosen to be distantly related (e.g., no siblings)."

Supplementary Figure 3

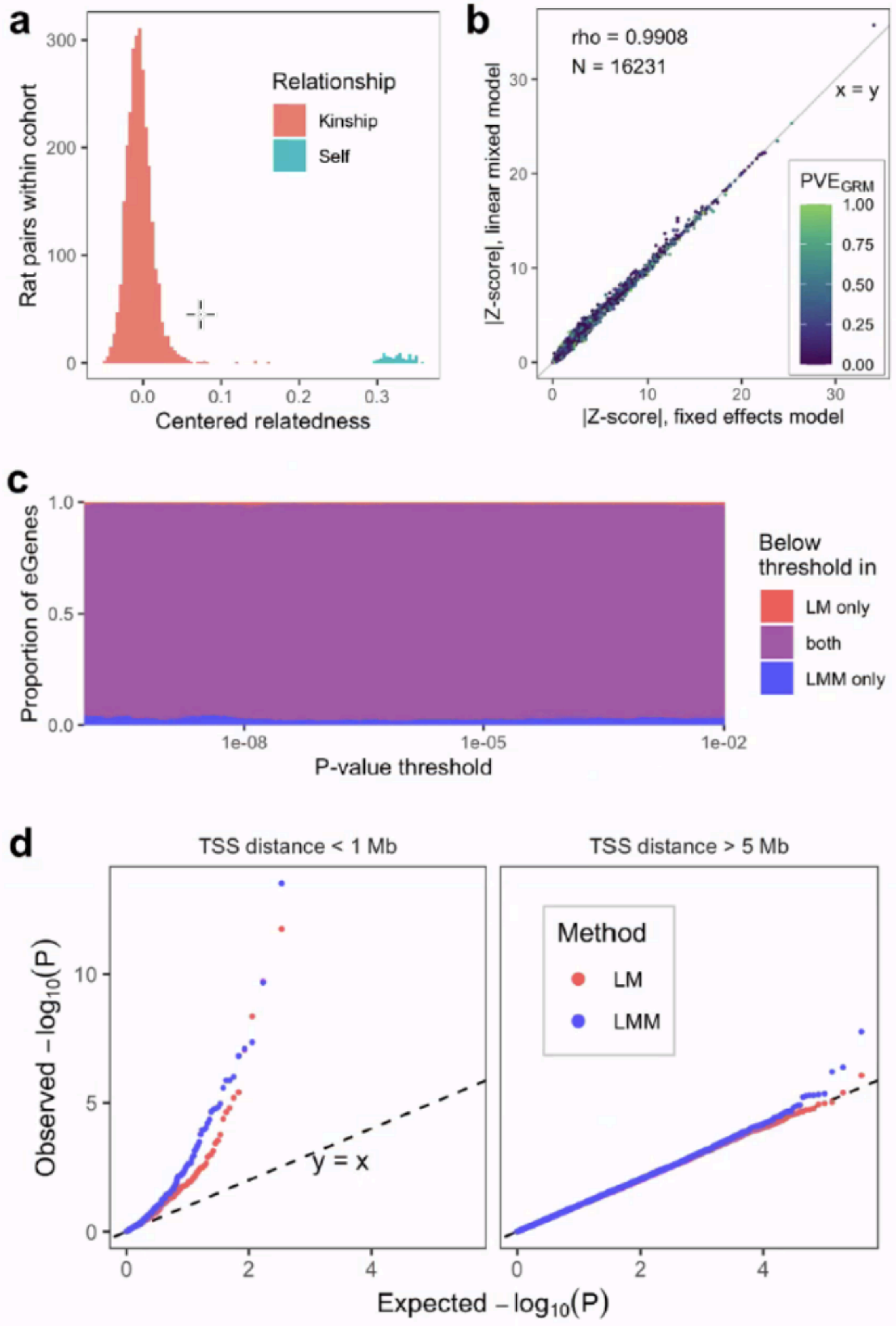

2. GRM construction for RatXcan associations: The manuscript does not explicitly state which variants are used in the GRM for RatXcan associations. (Apologies if I overlooked this information.) Are all available variants used, or are those within the ±1Mb window for each focal gene excluded? Alternatively, are LD-pruned variants employed? Using whole-genome LD-pruned variants for GRM construction seems a straightforward approach that could control inflation, despite potential proximal contamination.

Response: We used all genome-wide variants to construct the GRM. We were concerned that pruning for LD could reduce the effectiveness of correction. We did not remove variants in the proximity of the gene, nor did we apply a leaving-one-chromosome out (LOCO) approach because these components help us account for polygenic effects of SNPs in the region that do not act on the phenotype through the expression of the gene being tested (Liang et al 2023).

We now clarify in the results that the GRM is based on genome-wide SNPs, and that we did not apply LD-pruning: "*We also investigated the effect of pruning for LD before calculating GRM or removing variants in the proximity of the tested gene and found that they do not completely correct the inflation (see **Fig. S4** and **S5**). Hence, we recommend using genome-wide SNPs without pruning for LD or filtering out proximal SNPs.*"

3. Applicability of RatXcan vs. PrediXcan and S-PrediXcan: It appears that RatXcan is suitable for samples of highly related individuals, while PrediXcan is not. Could you clarify the applicability of S-PrediXcan in this context? This information would be particularly valuable for farm animal genetics researchers. Several GTEx projects for farm animal species (e.g., PMIDs: 35953587, 38177344) have used S-PrediXcan, which seems potentially inappropriate given that S-PrediXcan, like PrediXcan, is designed for samples of unrelated individuals. Considering that individuals in cattle or pig populations are often highly related due to strong artificial selection and extensive use of artificial insemination (elite male animals may have tens of thousands of offspring), there's a concern about the potential misuse of S-PrediXcan in these farm animal GTEx projects. A clarification of this issue in the Discussion section would be greatly appreciated.

Response: In general, we recommend running mixed effects models using genome-wide SNPs (RatXcan), which is simply a mixed effects TWAS, for sample sizes under ~50K. For larger samples, or for samples for which individual genotype data are not available, we now recommend using the inflation correction proposed in Liang et al 2023 rather than S-PrediXcan.

We have added these recommendations to the discussion:

"This mixed effects modeling approach implemented in RatXcan can be applied to human and other species TWAS when individual-level data are available. However, for biobank-scale data, we recommend using the summary statistics-based method in Liang et al. (2023). S-PrediXcan and other summary statistics-based methods that do not address the inflation driven by polygenicity and relatedness as described here and in Liang et al (2023) will yield higher false positive rates than expected."

"[...] our method depends on having access to individual-level data and needs to compute the GRM and the eigenvector decomposition, which may limit the application to medium sample sizes (under ~50K). For larger sample sizes, or for samples for which individual genotype data are not available, we recommend using the method in Liang et al. (2023), which can be applied using GWAS summary statistics."

Reviewer #2

In this manuscript, the authors extended the PrediXcan methodology to outbred heterogeneous stock (HS) rats. They developed RatXcan to account for close familial relationships among HS rats, trained transcript predictors for about 9,000 genes using reference genotype and expression data from five rat brain regions, and tested the association between predicted expression and body length and BMI in 5,401 densely genotyped HS

rats. The manuscript is well structured and clearly written. I have a few comments and suggestions for the authors, which I listed below.

1. I think eq 2b (page 17) was not really a standard linear regression model in the sense that the residual variance (\tilde\epsilon) was fixed as 1. Perhaps a better "decorrelation" approach would be to left multiply Y and T by (h^2GRM+(1-h^2)I)^(-1/2) which does not include \sigma^2, so that the residual variance in eq 2b can be estimated from a standard linear regression model.

Response: This is a very good suggestion. We have edited the description to make this point clearer. Now we define \Sigma = \sigma^2 * \Gamma and make it explicit that we use Gamma rather than Sigma for the decorrelation.

2. Were 88 HS rats in the training set assumed to be completely unrelated? It was unclear whether and how relatedness was adjusted for in the elastic net, and some clarifications would be helpful.

Response: Similar to our response 1 to reviewer 1, these rats are distantly related. Since the HS rats are bred in a laboratory, they are all part of an extended pedigree, as illustrated in Supplemental Figure 3 (reproduced above) of Munro et al (2022). The need to account for relatedness was investigated in that paper where it was concluded that it was not necessary as there were no substantive differences between the adjusting and not adjusting for relatedness. As noted above, we have added a sentence to the discussion and cited our prior work (Munro et al 2022) to justify this decision.

3. In addition to 0.1, 0.05, 0.01, it would be great to evaluate the type I error rates of RatXcan at much lower significance levels, since the method was applied to real data at the Bonferroni-corrected significance level of 0.05/8,272=6.04e-6.

Response: We have updated Figure 3D to include the Bonferroni-corrected significance threshold of 1e-6, and included the following to the results: *"To get a better estimate of the proportion of false positives when using 1e-6 as a threshold, we combined 2.9 million tests (5,879 genes by 100 simulations by 5 heritability values) and found 3 tests below the threshold, yielding a false positive rate of 1.02e-06 and further confirming the calibration of our corrected test."*

Minor:

1. Figure 2a legend showed R^2=0.72 and R=0.65. It could be a typo (R=0.85?). Also most p-values in Figure 2 legend were shown as P < 2.2e-16 which was not very informative. It would be nice to show lower p-values with a better precision.

Response: Done.

2. Figure 2c was not very informative. It was unclear whether "prediction performance" was defined using R^2 or R. It would be nice to add the data points in a scatter plot, in addition to the line and confidence band. Similarly, it would be nice to show heritability data points in Figure S2.

Response: Figure 2c was using R, not R^2, this has been clarified in Figure 2c and in the figure legend. We have now added Supplemental Figure S3 which shows the scatter plot.

3. In the second paragraph on page 11, (P=1.76e-6; P=3.62e-6) looked a little confusing. It would be better to place each p-value right after "BMI" and "body fat percentage" using two separate parentheses.

Response: Done.

4. Methods – Experimental model and subject details: "a prior study of 3,173 as well as more than 2,000 additional HS rats". Please give the exact number of "additional HS rats" if possible.

Response: Details now added *"a prior study of 3,173 (Chitre et al., 2020) as well as 2,228 additional HS rats"*

5. Methods – last paragraph on page 13: TPM should be defined.

Response: We have now defined TPM as transcripts per million.

Reviewer #3

In the manuscript under consideration, the authors presented RatXcan, an extension of their TWAS (PrediXcan) to outbred heterogeneous stock (HS) rats. The main innovation is to use a linear mixed model (eq1 on page 16 where a random effect term is added) to conduct the association mapping between predicted gene expressions and the phenotype in a typical TWAS protocol. By the addition of the random term, the infinitesimal polygenic effect is captured therefore the inflated p-value due to population structure (or uneven relatedness) is controlled. The LMM is solved by standard de-correlation procedure in which the data are transformed and then associated. The works looks intuitively sound, and the mathematical derivations are all correct for me. The real data analysis looks routine and solid for me as well. I am happy to endorse its publication. I have the following suggestions for the authors to consider.

Response: We thank the reviewer for the positive feedback.

An important concern is on the problem of not using LMM in PrediXcan back to their 2015 Nat Genet paper. Given the popularity of LMM (Kang et al 2010 Nat Genet; and a few others) in GWAS before the publication of PrediXcan, it is counterintuitive that PrediXcan does not adapt LMM in the first place, causing the massive inflation of p-values. The present work acts as a nice remedy to this problem. This is an inherent problem of all TWAS analysis for human cohorts where population structure is not avoidable, instead of just in HS rats. I feel that the authors may be explicit on this issue and explain that the current work may be used to replace PrediXcan (instead of just for rats).

Response: This is an excellent point and have now modified the introduction to clarify that this method is applicable to any species, including humans and other model organisms, where relatedness and population structure often need to be addressed: *"The utility of this mixed effects modeling goes beyond the rat data presented here and should be applicable to other species as well as to account for population structure in human data."*

The average heritability of gene expressions (Table 1) is way higher than it is in humans. Any evidence showing this estimate is correct from other literatures? From the materials section, I see that only 88 rats' data are available. Will this small sample size cause problem to an overestimate of expression heritability?

Response: Our rat cohorts are likely subjected to a more homogeneous environment than an equivalently sized human cohort, which will lead to higher heritability (i.e., smaller denominator driven by heritable and environmental factors). Another factor likely increasing our estimates of rat heritability is the relatedness of the HS rats, despite our effort to select distantly related ones. We added the following paragraph to the discussion section.

"We found higher heritability estimates for gene expression traits in rats compared to typical human studies, which could be due to the fact that our rat cohorts are likely to be subjected to a more homogeneous environment than an equivalently sized human cohort, which will lead to higher heritability (i.e., smaller denominator driven by heritable and environmental factors). Another factor likely increasing our estimates of rat heritability is the relatedness of the HS rats, despite our effort to select distantly related ones."

The work (Liang et al 2023) has been cited many times in the main text, however I can't find it in the references section. This actually serves as critical evidence for the claims of resolving p-value inflations. Nevertheless, I

tend to trust this work indeed provides the claimed evidence, as intuitively I agree with the authors' argument on this point.

Response: Thank you for bringing this to our attention. We have now included the missing citation (Liang Y, Nyasimi F, Im HK. On the problem of inflation in transcriptome-wide association studies. bioRxiv. 2023 Oct 20:2023.10.17.562831. doi: 10.1101/2023.10.17.562831. PMID: 37904952; PMCID: PMC10614931).

Have all data underlying the figures and results presented in the manuscript been provided?
Large-scale datasets should be made available via a public repository as described in the *PLOS Genetics* data availability policy, and numerical data that underlies graphs or summary statistics should be provided in spreadsheet form as supporting information.

Reviewer #1: Yes

Reviewer #2: Yes

Reviewer #3: Yes

PLOS authors have the option to publish the peer review history of their article (what does this mean?). If published, this will include your full peer review and any attached files.

If you choose "no", your identity will remain anonymous but your review may still be made public.

Do you want your identity to be public for this peer review? For information about this choice, including consent withdrawal, please see our Privacy Policy.

Reviewer #1: No

Reviewer #2: No

Reviewer #3: No

In compliance with data protection regulations, you may request that we remove your personal registration details at any time. (Remove my information/details). Please contact the publication office if you have any questions.
