# Supplementary material for: RatXcan: A framework for cross-species integration of genome-wide association and gene expression data
Source: PLoS Genet. 2025 Mar 31;21(3):e1011583. doi: 10.1371/journal.pgen.1011583 (PMC12052193; doi:10.1371/journal.pgen.1011583)
Supplement: Attachment — Submitted filename: response2reviewers-r2-ratxcan.docx [file pgen.1011583.s010.docx]

PGENETICS-D-24-00652R1

RatXcan: A framework for cross-species integration of genome-wide association and gene expression data

PLOS Genetics

**Reviewers' comments:**

Reviewer's Responses to Questions

**Comments to the Authors:
 Please note here if the review is uploaded as an attachment.**

Reviewer #1: The authors have adequately addressed my previous comments.

Reviewer #2: The authors have appropriately addressed most of my previous comments. A few remaining minor issues:
 1. There is a typo in the derivation of the covariance of \Gamma^(-1/2) (\mu+\epsilon) on page 17. The last line should be \Gamma^(-1/2) \sigma^2\Gamma \Gamma^(-1/2) = \sigma^2 I, and \sigma^2 is missing on both sides of the equation.

Done

2. Now the authors have added type I error rates at the significance level of 1e-6 in Figure 3d, but the figure legend has not been updated.

Done

3. The authors updated two p-values < 2.2e-16 in Figure 2 legend, but there are still three p-values showing P < 2.13e-30, P < 2.20e-16 and P < 2.20e-16 which I think are not very informative. Please show the small p-values with a better precision if possible.

Done

Reviewer #3: My previous comments are all addressed.
